# *ycf*1-*ndh*F genes, the most promising plastid genomic barcode, sheds light on phylogeny at low taxonomic levels in *Prunus persica*

**DOI:** 10.1186/s43141-020-00057-3

**Published:** 2020-08-14

**Authors:** Mohamed Hamdy Amar

**Affiliations:** Egyptian Deserts Gene Bank, Desert Research Center, B.O.P, Cairo, 11753 Egypt

**Keywords:** *P. persica*, Chloroplast, DNA barcode, *ycf*1-*ndh*F genes

## Abstract

**Background:**

Chloroplast genome sequencing is becoming a valuable process for developing several DNA barcodes. At present, plastid DNA barcode for systematics and evolution in flowering plant rely heavily on the use of non-coding genes. The present study was performed to verify the novelty and suitability of the two hotspot barcode plastid coding gene *ycf*1 and *ndh*F, to estimate the rate of molecular evolution in the *Prunus* genus at low taxonomic levels.

**Results:**

Here, 25 chloroplast genomes of *Prunus* genus were selected for sequences annotation to search for the highly variable coding DNA barcode regions. Among them, 5 genera were of our own data, including the ornamental, cultivated, and wild haplotype, while 20 genera have been downloaded from the GenBank database. The results indicated that the two hotspot plastid gene *ycf*1 and *ndh*F were the most variable regions within the coding genes in *Prunus* with an average of 3268 to 3416 bp in length, which have been predicted to have the highest nucleotide diversity, with the overall transition/transversion bias (*R* = 1.06). The *ycf*1-*ndh*F structural domains showed a positive trend evident in structure variation among the 25 specimens tested, due to the variant overlap^’^s gene annotation and insertion or deletion with a broad trend of the full form of IGS sequence. As a result, the principal component analysis (PCA) and the ML tree data drew an accurate monophyletic annotations cluster in *Prunus* species, offering unambiguous identification without overlapping groups between peach, almond, and cherry.

**Conclusion:**

To this end, we put forward the domain of the two-locus *ycf*1*-ndh*F genes as the most promising coding plastid DNA barcode in *P. persica* at low taxonomic levels. We believe that the discovering of further variable loci with high evolutionary rates is extremely useful and potential uses as a DNA barcode in *P. persica* for further phylogeny study and species identification.

## Background

Peach [*Prunus persica* (L.) Batsch], a member of the Rosaceae family, is one of the most genetically important fruit trees in temperate regions [1]. It belongs to five wild relatives which are generally accepted: *Prunus mira*, *Prunus davidiana* Franch, *Prunus davidiana* var. potaninii Rehd., *Prunus kansuensis* Rehd., and *Prunus ferganensis* Kost. and Riab [2]. These wild relatives have attracted attention because they allow a natural diversity panel that offered an opportunity to introduce the traits of interest from native species into the peach texture. Owing to the high similarity and monophyletic clades concept within *P. persica* may cause a lot of complications in the classification of the species. Therefore, with the recent progress toward the whole genome sequence of peach [3] along with the assembly of Chloroplast DNA (cpDNA) genome data of *P. persica* cv. Lovell [4], provided a new era for the development of comparative cpDNA genome studies and the discovery of DNA barcode genes in peach. Fortunately, the decreasing cost of high-throughput sequencing of the cp genome offers opportunities to gain more cp genome sequences and discover useful particular DNA barcode of coding and non-coding plastid genes in *Prunus* species [5].

It is well known that the chloroplasts are the photosynthetic organelles in plant cells, provide energy to green plants and plays an important role in sustaining life [6]. The chloroplast genome is the third-largest genome after the nuclear genome and mitochondria, which encodes many key proteins that are involved in photosynthesis and other metabolic processes [7]. The chloroplast genomes have different features, e.g., maternal inheritance in most angiosperms, and high conservation in genome structure and gene contents [8]. The cpDNA genome is extremely conserved with a self-replicating circular molecule and has a typical quadripartite structure, in which two inverted repeats (IRs) are separated by a large single-copy region (LSC) and a small single-copy region (SSC) [9]. Most of the cpDNA contains approximately 110–133 genes, including protein-coding genes (CDS), ribosomal RNA genes, and transfer RNA genes. The non-coding and coding regions of the chloroplast genome had a diverse signature at both high and low taxonomic levels, making them appropriate for systematic and evolution studies [10]. Albeit, the non-coding region has less functional constraint than the coding region, it offers superior levels of evolutionary rate for phylogenetic and barcoding studies at the subspecies level, while the coding region is highly conserved and suitable only for higher taxonomic levels [11].

In the past decade, the two protein-coding genes *mat*K and *rbc*L were chosen as core plant DNA barcodes [12], while other protein-coding genes, like *atp*F-H, *psb*K-I, *rop*C1, and *rpo*B, are lacking resolution and have been recommended as supplemental barcodes in diversity within flowering plants [13]. Unfortunately, the discrimination power of discovered barcodes coding genes is too weak to drive through all species, especially in higher plants [14]. Hence, there are no universal barcode loci neither for all plants nor for *Prunus* species. Dong et al. [12] proposed that *ycf*1 is the most promising plastid DNA barcode for land plants and plays an important role in genome evolution. Other evidence supposed that among the protein-coding genes, *ycf*1 and *ndh*F are appreciated sources of phylogenetic relationship provide effective information and DNA barcodes-based cpDNA genome for phylogeny and species identification in breeding resources [15–17]. Later, Jeon and Kim [9] suggested that the combination of two-locus *ycf*1 and *ndh*F genes is beneficial for deciphering phylogenetic relationships between closely related taxa in Rosaceae. Although these two hot spot genes have superior efficiency in discrimination at the low taxonomic level, still little attention for DNA barcoding and molecular evolution purposes are received [12, 18].

To date, with the documented deficiency of the *ycf*1-*ndh*F coding genes in *Prunus*, we reported for the first time a detailed overview of these hotspot regions to investigate evolutionary relationships between 25 *Prunus*, *Malus*, and *Pyrus* species. Through this research, the performance and efficiency of *ycf*1*-ndh*F genes were evaluated as hotspot regions for DNA barcoding and biodiversity which may be helpful in future breeding programs of peach. For this purpose, we achieved a comparative structure variation, the overlapping of *ycf*1*-ndh*F genes sequences, and phylogeny analysis within the species level of the ornamental, cultivated, and wild haplotype of *P. persica*.

## Methods

### Plant materials and DNA extraction

Peach specimens (*P. persica*) used in this study contained three edible cultivars, one ornamental cultivar, and a wild relative *P. mira* (Table 1). These five specimens were collected from the field gene bank of Chinese Academy of Sciences (CAS), Wuhan, China, in the juvenile stage in the spring season. Total genomic DNA was extracted from 100 mg of fresh leaves using Plant Genomic DNA Extraction Kit (DP305-03, Tiangen Biotech, Beijing, China) according to the manufacturer’s instructions. The DNA quantity was assessed using a spectrophotometer (Nanodrop 2000, Thermo Fischer, USA). Both the stock and diluted portions were stored at – 80 °C until use.

**Table 1:** List of taxa sampled in this study with the gene length, position, overlapped, and intergenic regions

		*Ycf*1 gene			*ndh*F gene					
Sp. code	Species Name	Start (bp)	End (bp)	Gene Length (bp)	Start (bp)	End (bp)	Gene length (bp)	Overlapped (bp)	IGS (bp)	Full length *ycf*1-*ndh*F (bp)
1	*Pyrus pyrifolia*	113121	114194	1073	114195	116438	2243	–	–	3316
2	*Malus prunifolia*	112903	113976	1073	113977	116220	2243	–	–	3316
3	*Pyrus spinosa*	113405	114478	1073	114479	116722	2243	–	–	3316
4	*P. persica*	111350	112417	1067	112410	114635	2225	124	–	3416
5	*P. maximowiczii*	111213	112274	1061	112251	114482	2231	–	9	3301
6	*P. serrulata*	111238	112293	1055	112303	114528	2225	–	9	3289
7	*P. subhirtella*	111320	112375	1055	112385	114610	2225	116	–	3396
8	*P. yedoensis*	111292	112377	1085	112362	114590	2228	–	16	3329
9	*P. mongolica*	111209	112264	1055	112281	114506	2225	–	59	3339
10	*P. dulcis*	111747	112808	1061	112868	115099	2231	109	–	3401
11	*P.davidiana*	111176	112222	1046	112214	114445	2231	109	–	3386
12	*P. mume*	111579	112625	1046	112617	114848	2231	109	–	3386
13	*P. kansuensis*	111175	112221	1046	112213	114444	2231	124	–	3401
14	*P. yedoensis*	111091	112152	1061	112129	114360	2231	116	–	3408
15	*P. pseudocerasus*	111318	112403	1085	112388	114616	2228	–	9	3322
16	*P. humilis*	111277	112332	1055	112342	114567	2225	–	23	3303
17	*P. serotina*	111439	112485	1046	112510	114708	2198	–	24	3268
18	*P. pedunculata*	112548	113585	1037	113610	115835	2225	109	–	3371
19	*P. tomentosa*	111410	112456	1046	112448	114679	2231	124	–	3401
20	*P. takesimensis*	111938	112999	1061	112976	115207	2231	108	–	3400
21	(CJX) Cultivar (*P. persica*)	111182	112228	1046	112220	114451	2231	109	–	3386
22	(CDH) Cultivar (*P. persica*)	111208	112269	1061	112246	114477	2231	124	–	3416
23	(CMJ) Cultivar (*P. persica*)	111214	112275	1061	112252	114480	2228	124	–	3413
24	(OMT) Ornamental (*P. persica*)	111200	112261	1061	112238	114469	2231	124	–	3416
25	(WGH) (*P. mira*)	111182	112228	1046	112220	114451	2231	109	–	3386

### DNA sequencing, genome assembly, and validation

The Illumina HiSeq 2500 platform was used to sequence the total DNA of the five studied specimens. After sequencing, the raw data was initially screened to remove low-quality regions affecting the data quality and subsequent analysis needed to obtain the expected clean data. The cpDNA genome was assembled by mapping onto the public complete chloroplast genome of *P. persica* cv. Lovell (GenBank accession HQ336405) [4], and the genome assembly and alignment analyses were performed using Geneious R10 program (http://www.geneious.com; Biomatters Ltd., Auckland, New Zealand).

### Genome annotation and analysis

In the present study, 25 cpDNA genomes were used for annotation, including the 5 peach specimens from our materials, in addition to the 20 cpDNA genomes that were downloaded from NCBI GenBank database. However, *Pyrus pyrifolia*, *Pyrus spinosa*, and *Malus prunifolia* were used as outgroup. These 25 cpDNA genomes representatives all major indigenous of *Prunus* species. Gene annotation of the 25 cpDNA genomes was performed with the online program Dual Organellar GenoMe Annotator (DOGMA) [19]. Initial annotation, putative starts, stops, and intron positions were determined, and then the draft annotation was inspected and corrected manually by comparison with a homologous gene with the chloroplast genome of *P. persica* (NC_014697) from the NCBI database.

### Identification *of ycf*1*-ndh*F genes, sequence editing and alignment

The *ycf*1-*ndh*F genes were obtained from the 25 cpDNA genomes using DOGMA analysis to compare the structure sequence, and the multiple sequence alignment was done using MUSCLE v3.70+ fix1-2 [20], and manually adjusted, as necessary. Nucleotide diversity (π), estimated values of transition/transversion bias (*R*), and nucleotide substitutions (*r*) for each sequence were performed using MEGA X program [21].

### Phylogenetic inference

The analysis of the consensus phylogenetic tree was performed using 25 nucleotide sequences of *ycf*1-*ndh*F genes, including 22 species of *Prunus* in addition to the 3 species for *Pyrus* and *Malus* as an outgroup (*Pyrus pyrifolia*, *Pyrus spinosa*, and *Malus prunifolia*). To gain accurate perspectives on genetic diversity, a graphic demonstration of principal component analysis (PCA) was carried out to display the multi-dimensional genetic relationship and its partition among specimens using the ClustVis web tool for visualizing clustering of multivariate data [22]. The evolutionary history was inferred by using the maximum likelihood method (ML) based on the Tamura-Nei model [23]. The maximum likelihood (ML) tree was computed using MEGA X software. The bootstrap consensus tree inferred from 1000 replicates was taken and searched for the best-scoring ML tree simultaneously to represent the evolutionary history of the 25 specimens tested.

## Results

### Performance of *ycf*1 and *ndh*F genes identifications

**Fig. 1:**
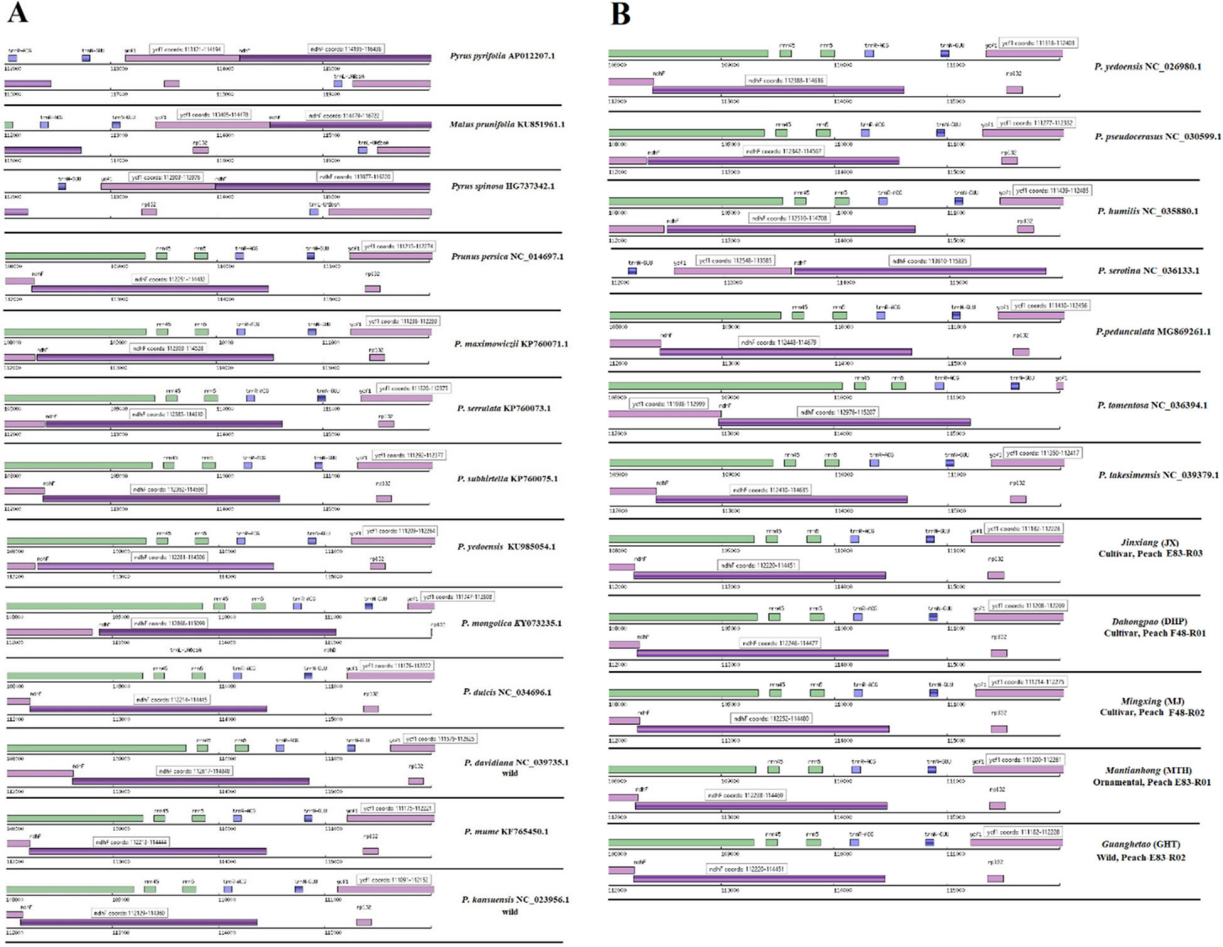
Visualization of *ycf*1-*ndh*F genes within 25 species in our study using Dual Organellar GenoMe Annotator (DOGMA) where the putative starts, stops, and intron positions were annotated

At first, to empirically test the regions identified as most appropriate for barcoding in the plastid coding genes of *P. persica*, automatic genome annotations were performed among the 25 cpDNA genomes of Rosaceae (Fig. 1). According to original gene annotations analysis and our previous data on this material (data not published), several invariable loci in the analyses were ignored due to inadequate identification, e.g., *rbc*L, *mat*K, *ndh*A, *ycf*2, *ycf*3, *rop*C1, *rpo*C2, *rpo*B, *rps*16, *clp*P, *psb*B, *atp*F, *atp*A, trnK-UUU, and *trn*H-*psb*A (data not shown). To circumvent the challenges related to a single-locus approach, this study undertook a two-locus analysis with its overlapping or intergenic spacer (IGS) and insertion/deletion as a useful option in delineating closely related peach sequence variations based on the combination of complete two protein-coding genes *ycf*1-*ndh*F of the chloroplast genome.

**Fig. 2:**
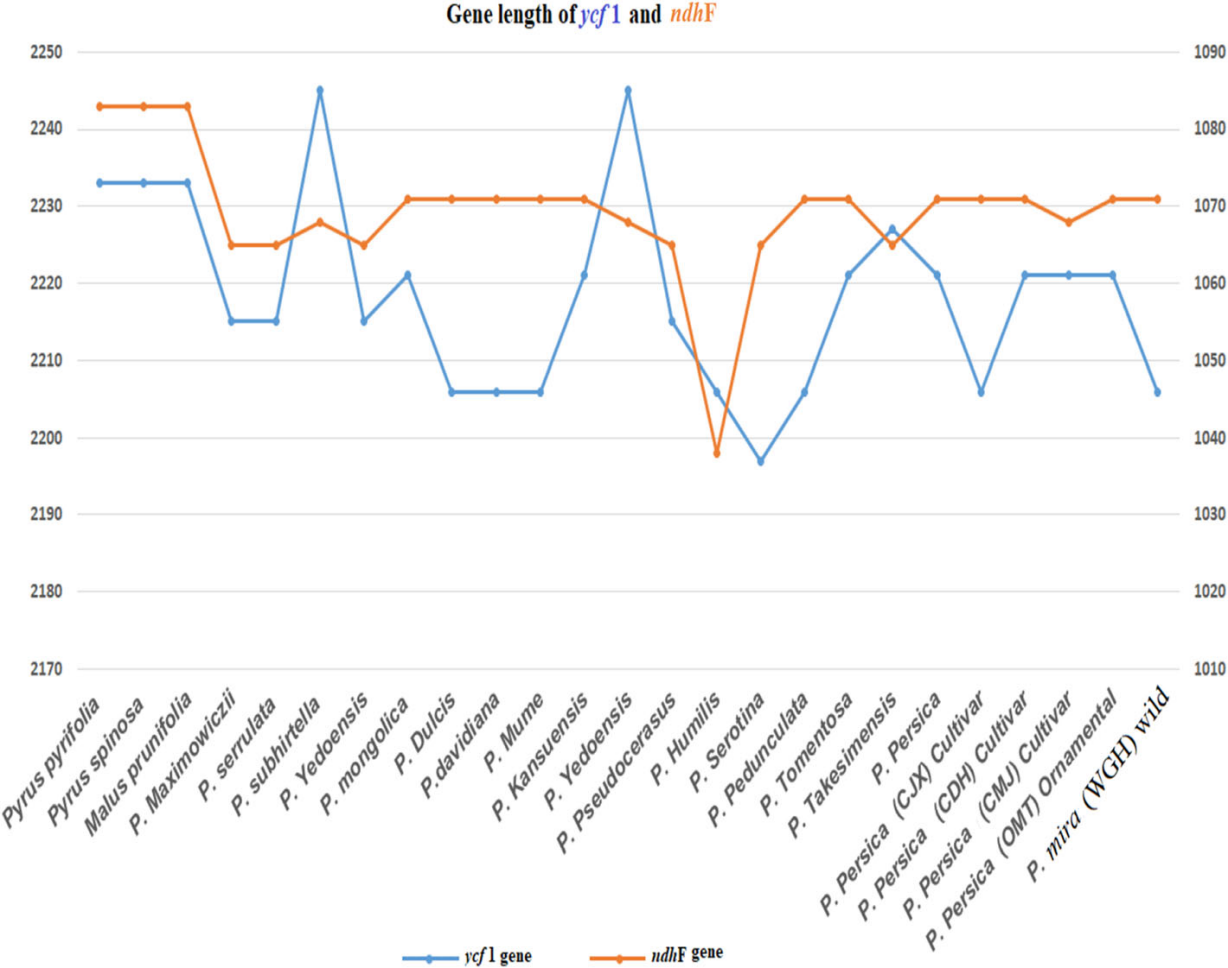
Diagram represented a comparison gene length of *ycf*1 and *ndh*F among the 25 specimens tested in this study

The position of *ycf*1 in IRA regions varied from 1037 to 1085 bp (Table 1 and Fig. 2). It is worth noting that the ornamental and cultivated species in our study so-called OMT, CMJ, CDH, and CJX gave similar length with the *P. persica* and *P. Kansuensis* of 1061 bp, while a slight lower size variation was observed in the two wild types *P. davidiana* and *P. mira* with 1046 bp. By comparison, *ndh*F gene had a much higher position in SSC regions varied from 2098 to 2234 bp (Table 1 and Fig. 2). However, all cultivated, wild type, and ornamental species in our sampling had different *ndh*F gene length harboring 2231 bp, except for CMJ cultivar which had a slightly lower sequence length with 2228 bp. Overall, the two-loci *ycf*1*-ndh*F ranged from 3268 to 3416 bp in length, showed great sequence variation than the single-locus approach due to the variant overlaps of gene annotation and intergenic regions.

### Overlapped and intergenic sequences within *ycf*1 and *ndh*F genes

**Fig. 3:**
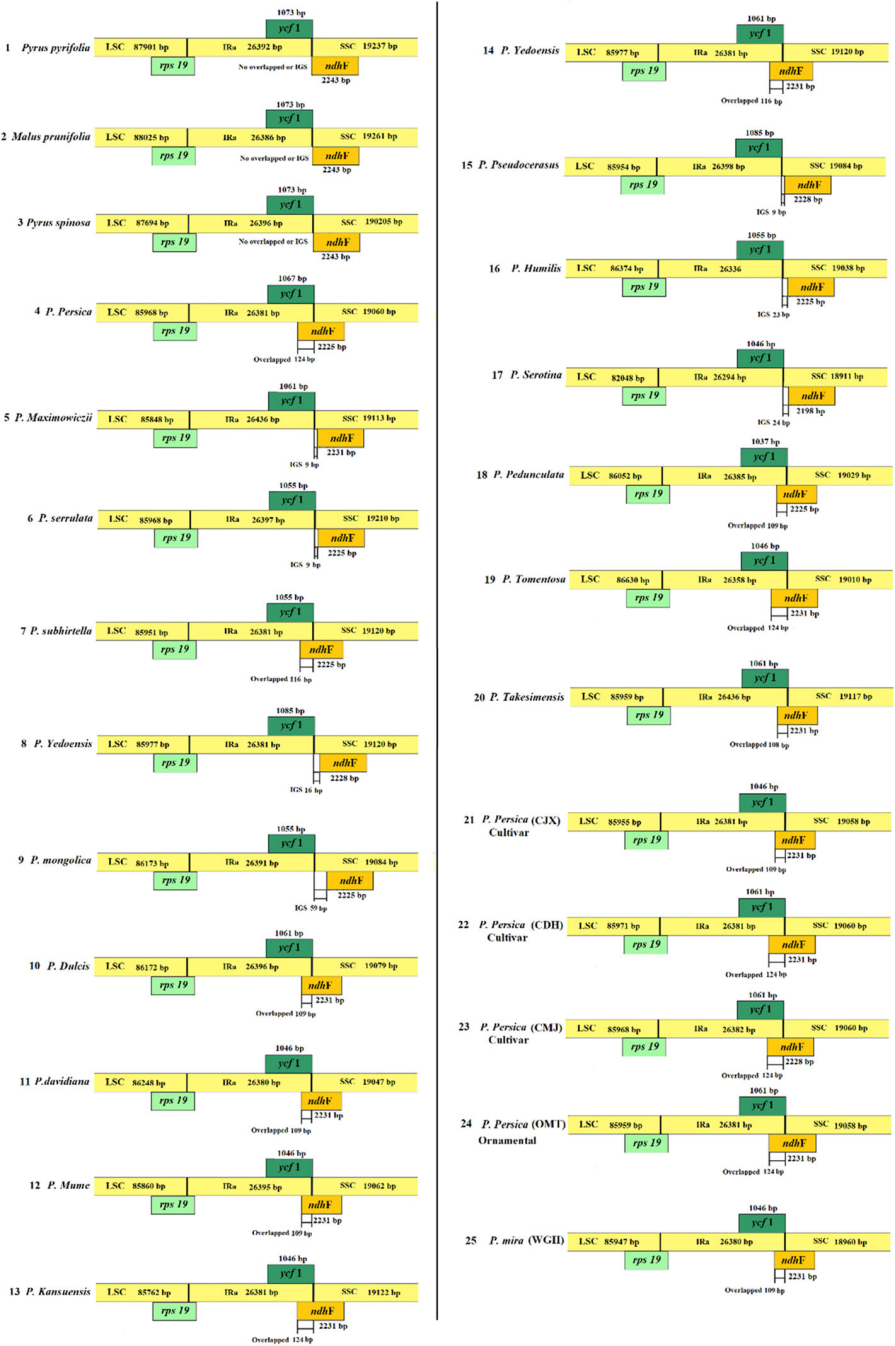
Distance between adjacent and junctions of *ycf*1-*ndh*F genes among 25 specimens tested with the relative changes at or near the IRa/SSC borders. In each lane, boxed ribbons show the overlapped and the intergenic region (IGS) with the total lengths

In an in-depth look, another remarkable difference identified the overlapped phenomenon and intergenic sequence region in the IRA/SSC border within *ycf*1-*ndh*F genes of the plastid genome. The border between four junctions usually differs among plants showed a slight variation in size among the 25 tested specimens (Fig. 3). As a result, the pseudogenes, *ycf*1 gene present at the IRa/SSC border and is partially located inside the IR region. Among the 25 specimens tested, only 15 showed a variant overlapped region between *ycf*1 and *ndh*F genes with the size ranging from 109 to 124 bp (Table 1). By contrast, the intergenic region was identified within only 7 specimens which harbored IGS sequence ranged from 9 to 59 bp (Table 1). Among all, *P. mongolica* showed the highest IGS sequence variation harbored 59 bp, followed by *P serotina*, *P. humilis*, and *P. yedoensis* with 24, 23, and 16 bp, respectively. While the three specimens *P. maximowiczii*, *P. serrulata*, and *P. pseudocerasus* contained the lowest IGS with 9 bp in length. By contrast, the rest three specimens of *Pyrus pyrifolia, Pyrus spinosa*, and *Malus prunifolia* showed the opposite trend with no intergenic region or overlapped ones. Taken together, the two-locus *ycf*1-*ndh*F structural domains demonstrated divergence evident in structure variation among the 25 tested specimens.

### Sequence divergence of *ycf*1-*ndh*F genes

To obtain a comprehensive knowledge on the *ycf*1 and *ndh*F sequence divergence among taxa, the averages of nucleotide frequencies were A (33.95%), T/U (33.82%), C (16.12%), and G (16.11%) with an average of AT (33.92%) and GC (16.08%) contents (Table 2). In order to determine the transition/transversion bias (R), the nucleotide substitution pattern was estimated to describe the superior substitution pattern using Kimura 2-parameter analysis with five models (T92+G+I, HKY+G+I, GTR+G+I, TN93+G+I, and K2+G+I). The highest rate of substitutions values (*r*) for each nucleotide pair was detected in *r* (GA ± 0.19) and *r* (CT ± 0.018), revealing high levels of substitutions. By contrast, the lower values of substitution were observed within *r* (AC; GC; CG; TG ± 0.04), respectively (Table 3). Furthermore, the transition/transversion rate ratios, recorded a higher transition/transversion rate for purine (K1 = 2.57) compared to the transition/transversion rate for pyrimidine (K2 = 2.28). While the overall transition/transversion bias is *R* = 1.06, which gives support for the dominance of the transitions over transversion in peach germplasm.

**Table 2:** Maximum composite likelihood estimate of the pattern of nucleotide substitution among 25 different nucleotide sequences

	A	T	C	G
A	*–*	*7.64*	*3.64*	**9.36**
T	*7.67*	*–*	**8.31**	*3.64*
C	*7.67*	**17.43**	*–*	*3.64*
G	**19.73**	*7.64*	*3.64*	*–*

Where each entry shows the probability of substitution (*r*) from one base (row) to another base (column). Rates of different transitional substitutions are shown in bold and those of transversionsal substitutions are shown in italics. Against the nucleotide frequencies are 33.95% (A), 33.82% (T/U), 16.12% (C), and 16.11% (G). The transition/transversion rate ratios are K1 = 2.573 (purines), K2 = 2.282 (pyrimidines), and the overall transition/transversion bias is *R* = 1.06 with a total of 7110 positions in the final dataset

**Table 3:** Maximum likelihood fits using the Kimura 2-parameter model among 25 different nucleotide sequences for the combined locus of *Ycf*1-*ndh*F genes

Model	Invariant(+I)	R	Freq(A)	Freq(T)	Freq(C)	Freq(G)	*r*(AT)	*r*(AC)	*r*(AG)	*r*(TA)	*r*(TC)	*r*(TG)	*r*(CA)	*r*(CT)	*r*(CG)	*r*(GA)	*r*(GT)	*r*(GC)
T92+G+I	0.66	1.18	0.339	0.339	0.161	0.161	0.072	0.034	0.093	0.072	0.093	0.034	0.072	0.195	0.034	0.195	0.072	0.034
HKY+G+I	0.66	1.18	0.34	0.338	0.161	0.161	0.072	0.034	0.093	0.072	0.093	0.034	0.072	0.195	0.034	0.195	0.072	0.034
GTR+G+I	0.66	1.19	0.34	0.338	0.161	0.161	0.057	0.044	0.094	0.057	0.089	0.038	0.093	0.186	0.031	0.199	0.08	0.031
TN93+G+I	0.66	1.18	0.34	0.338	0.161	0.161	0.072	0.034	0.096	0.072	0.09	0.034	0.072	0.188	0.034	0.202	0.072	0.034
K2+G+I	0.69	1.71	0.25	0.25	0.25	0.25	0.046	0.046	0.158	0.046	0.158	0.046	0.046	0.158	0.046	0.158	0.046	0.046

*I* against evolutionarily invariable, *R* revealing estimated values of transition/transversion bias, *Freq* nucleotide frequencies, and *r* substitutions for each nucleotide pair

### Principal component analysis and phylogenetic inference

**Fig. 4:**
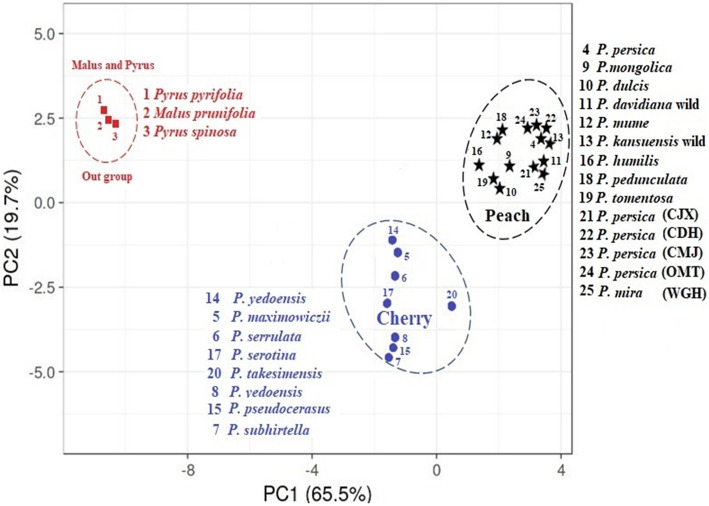
Schematic representation the principal component analysis (PCA) of 22 species of *Prunus* and 3 species of *Pyrus pyrifolia*, *Pyrus spinosa*, and *Malus prunifolia* as outgroup, while PC1and PC2 refer to the first and second principal component, respectively

Both PCA as well as a phylogenetic tree take a sequence data matrix as input where multiple dimensions of *ycf*1-*ndh*F genes region data are measured in multiple observations. The PCA plot data as presented in Fig. 4 formed three relatively clustered groups, with the total molecular variation of 65.5% and 19.7%, respectively. The cluster I compressed all ornamental, wild types, and cultivated specimens of peach and almond together with a closer relationship in a particular group, while cluster II assembled jointly all eight members of cherry species in the individual group. Meanwhile, the three species of *Pyrus pyrifolia*, *Pyrus spinosa*, and *Malus prunifolia* were separated individually as outgroup near to the PC2 axis.

**Fig. 5:**
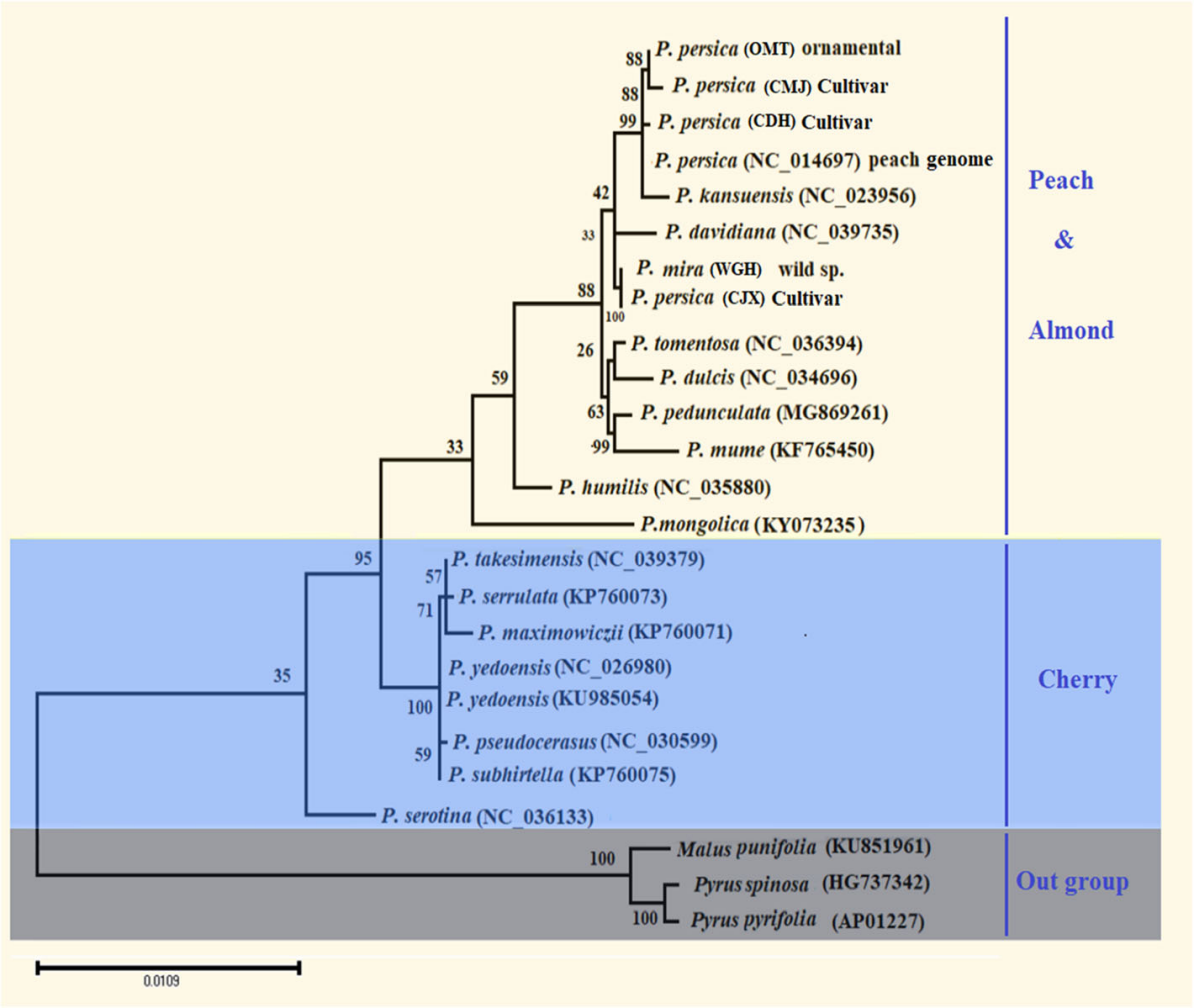
Phylogenetic trees of 22 species within genus *Prunus* and three species of *Pyrus pyrifolia*, *Pyrus spinosa*, and *Malus prunifolia* as outgroup. The entire sequence dataset was analyzed using maximum likelihood (ML), species group and outgroup are highlighted by a colorful background, and the scale bar shown on the bottom illustrates the relative genetic variability

To ensure the exact relationship between the 25 specimens tested, the phylogenetic tree was constructed based on the ML tree (Fig. 5). All the 22 *Prunus* specimens were classified into 3 major clades with highly bootstrap value within the peach, almond, and cherry groups. The three *P. persica* cultivars, OMT, CMJ, and CDH, were clustered with *P. persica* cv. Lovell formed a monophyletic clade and gathered into a common clade with *P. kansuensi* and *P. davidiana*, while *P. mira* and CJX cultivars were located in the basal position of the first clade confirming a close genetic relationship to peach. Furthermore, all almond and plum species were excluded together in the second monophyletic clade with a high proportion of joint relationship to the peach clade. The cherry group was further divided into two monophyletic groups. This suggested that there is great genetic diversity within cherry. A unifying clade, clade three, comprised the roots of six members of cherry species combining *P. takesimensis*, *P. serrulate*, *P. maximowiczii*, *P. yedoensis*, *P. pseudocerasus*, and *P. subhirtella*, while a black cherry (*P. serotina*) was placed independently in the basal position of the cherry clade. By contrast, *Pyrus pyrifolia, Pyrus spinosa*, and *Malus prunifolia* were shared individually as an outgroup of the tree. Herein, our results imply that peach underwent a domestication event that separated the cultivated peach from the wild species and cherry.

## Discussion

In recent years, attention has been paid to the advent of high-throughput sequencing. This technology offers opportunities to gain a more suitable plastid DNA barcode for flowering plants through the comparative analysis of full cp DNA genomes [7]. However, at lower taxonomic levels of flowering plants, the problem is that most of the chloroplast coding region genes have insufficient sequence variation rather than the non-coding regions to resolve inter- and intraspecific relationships [18]. We, therefore, turned our attention to two-loci coding plastid genes *ycf*1 and *ndh*F coding genes than expected would also meet the criteria needed for maximum utility as a coding hotspot locus in *Prunus*. Several earlier articles have been proposed that *ycf*1 and *ndh*F are useful information for DNA barcode and subject to positive selective pressure due to high variability [15, 18, 24, 25]. According to the interpretation of the recent plastid data in genus Rosa [9], *ycf*1 gene has a conversion of the 543th amino acid, while a frameshift mutation was found in the 3′ regions of *ndh*F genes with a higher substitution rate (R), resulting in numerous conservative and missense mutation. Mainly, there is extremely low-genetic variation due to the decrease of the substitution rate within the genus [26]. As it is well known, this difference occurs because substituting a single-ring structure for another single-ring structure is more likely than substituting a double ring for a single ring [27]. Here, our results showed a higher transition/transversion (R) rate in the DNA sequence variation with transitions occurred more frequently than transversions. Such variance among the rate of transition and transversion is a foundational principle for studies of molecular phylogeny [28].

Another striking characteristic is the overlapped phenomenon between the *ndh*F-*ycf*1genes; this is because of an unequal size variation or absence of overlapping in the expansion and contraction of the IR region [16], which indicated fast-evolving events. Previous results in Rosaceae [15] highlight the sizes of overlapped change from 110 bp in *P. pyfifolia* to 96 bp in *P. persica* and with 40 bp in *P. rupicola*. Our data infer a similar feature with obvious differences was observed ranged from 109 to 124 bp, especially between cultivated and wild types. This concept has gained much acceptance and support through recently plastid genome studies [9, 17, 29, 30].

With more direct interest in our results, a positive association in the IGS region was observed within the two genes, owing to sequence divergence in the cpDNA. Since the border of the IR region of cp genomes occasionally harbor insertion or deletion with a broad trend of IGS sequence, this might have led to higher sequence divergence in this region [30]. It has been well known that the non-coding regions are mostly responsible for the cpDNA genome size variation [11], thus, providing superior levels of variation and may be valuable for developing DNA barcodes to estimate phylogeny at a subspecies level [7, 31].

Peach taxonomy and phylogeny are often the subjects of controversy and the major obstacle in peach breeding. To verify the sensitivity of our phylogenetic tree, we compared our results with the recent genome evolution study [8]. During the evolutionary history of a certain lineage, we believe that one of the controversial issues raised in *P. persica* species is the relationship between cultivated and wild taxa. At present, the wild peach germplasm can offer many useful genes for peach improvement. Evidence suggested that *P. mira* is the oldest progenitor of peach [2], and it is considered ideal wild peach germplasm for improving cultivated peach plants [32]. It is worth noting that the ornamental cultivar is phylogenetically closely related to the edible cultivar, which supports the previous finding that most of the ornamental peach cultivars originated directly from *P. persica* [33]. As seen in our chloroplast phylogenomic tree, the cultivated peach species were derived from the three wild species presented in this study, *P. kansuensis*, *P. davidiana*, and *P. mira*. However, *P. kansuensis* shows a closer relationship with peach cultivars than *P. mira*. This is consistent with the previous genome resequencing analysis [2, 8], which supported the view that *P. kansuensis* is closer to *P. persica* than *P. mira* and *P. davidiana*. Several scholars have pointed out that *P. davidiana* is more primitive than *P. kansuensis*. This trend was supported by earlier evolutionary of genome re-sequencing in peach [8], which reveals that compared to *P. davidiana* and *P. dulcis*, there are increased inbreeding levels in the three-peach species (*P. persica*, *P. kansuensis*, and *P. mira*). Our data assume *P. mira* as the most closely related to *P. mongolica* and *P. dulcis*, and support the hypothesis of the hybrid origin of peach with almond [3, 34]. Furthermore, the current analyses strongly support the monophyly of *P. pseudocerasus* as the rootstock for Chinese cherry species [35].

Under the constraint background, *ycf*1-*ndh*F genes recover relationships among *Prunus* including peach, almond, and cherry, which have a taxonomic group with extremely poor sequence divergence. We believe that discovering further variable coding loci with high evolutionary rates is extremely useful and potential to be used as a coding DNA barcode in *P. persica* at low taxonomic levels. We tentatively put forward this study that might draw the attention of other scientists who have been working on assessing the evolutionary relationships among peach species.

## Conclusion

With the rapid progress of NGS technologies, a large number of cpDNA genome sequences have been developed during the last two decades, which is beneficial for genome evolution and developing several DNA barcodes in plants. The present study highlighted to check the resolution and sensitivity of two DNA barcoding hotspot locus *ycf*1 and *ndh*F genes, which can offer a new approach to resolve the phylogeny and systematics for closely associated species in *Prunus*. Noteworthy, our results revealed that the two-locus *ycf*1-*ndh*F was varied from 3268 to 3416 bp in length. We obtained a great sequence variation in the two-locus compared to the single-locus approach due to the significant structure variation, overlaps gene annotation, and intergenic regions. Collectively, our results of the PCA and the phylogenetic tree analysis indicate that accurate monophyletic annotations clade offer obvious classification without overlapping clusters between peach, cherry, and almond. The current study, therefore, recommends the usage of the two barcoding hotspot locus *ycf*1 and *ndh*F genes approach in delineating the *Prunus* genus at the varietal level and species identification.

## Data Availability

The datasets used and/or analyzed during the current study are available from the corresponding author on reasonable request.

## Abbreviations

cpDNA: Chloroplast DNA
IRs: Inverted repeats
LSC: Large single-copy region
SSC: Small single-copy region
CDS: Protein-coding genes
CAS: Chinese Academy of Sciences
DOGMA: Dual Organellar GenoMe Annotator
PCA: Principal component analysis
ML: Maximum likelihood
IGS: Intergenic spacer
R: Transition/T

## References

[1] Arus P, Verde I, Sosinski B, Zhebentyayeva T, Abbott AG (2012). The peach genome. Tree Genet Gen.

[2] Cao K, Zheng Z, Wang L, Liu X, Zhu G, Fang W, Cheng S, Zeng P, Chen C, Wang X, Xie M (2014). Comparative population genomics reveals the domestication history of the peach, *Prunus persica*, and human influences on perennial fruit crops. Genome Biol.

[3] Verde I, Abbott AG, Scalabrin S, Jung S, Shu S, Marroni F (2013). The high-quality draft genome of peach (*Prunus persica*) identifies unique patterns of genetic diversity, domestication and genome evolution. Nat Genet.

[4] Jansen RK, Saski CA, Lee S, Hansen AK, Daniell H (2010). Complete plastid genome sequences of three rosids (*Castanea*, *Prunus*, *Theobroma*): evidence for at least two independent transfers of rpl22 to the nucleus. Mol Biol Evol.

[5] Khan A, Asaf S, Khan AL, Al-Harrasi A, Al-Sudairy O, AbdulKareem NM, Khan A, Shehzad T, Alsaady N, Al-Lawati A, Al-Rawahi A (2019). First complete chloroplast genomics and comparative phylogenetic analysis of *Commiphora gileadensis* and *C. foliacea: Myrrh* producing trees. PLoS One.

[6] Douglas SE (1990). Plastid evolution: origins, diversity, trends. Curr Opin Genet Dev.

[7] Daniell H, Lin CS, Yu M, Chang WJ (2016). Chloroplast genomes: diversity, evolution, and applications in genetic engineering. Genome Biol.

[8] Yu Y, Fu J, Xu Y, Zhang J, Ren F, Zhao H, Tian S, Guo W, Tu X, Zhao J, Jiang D (2018). Genome re-sequencing reveals the evolutionary history of peach fruit edibility. Nat Commun.

[9] Jeon, J.H., and Kim, S.C., (2019). Comparative Analysis of the complete chloroplast genome sequences of three closely related East-Asian wild roses (Rosa sect*. Synstylae*; Rosaceae). Genes 10:23 10.3390/genes10010023 PMC6356658 30609873

[10] Li Y, Zhang J, Li L, Gao L, Xu J, Yang M (2018) Structural and comparative analysis of the complete chloroplast genome of *pyrus hopeiensis*—“wild plants with a tiny population”—and three other *pyrus* species. Int J Mol Sci 19(10):p.3262 10.3390/ijms19103262 PMC6214102 30347837

[11] Pervaiz T, Sun X, Zhang Y, Tao R, Zhang J, Fang J (2015). Association between Chloroplast and Mitochondrial DNA sequences in Chinese *Prunus* genotypes (*Prunus persica, Prunus domestica,* and *Prunus avium*). BMC Plant Biol.

[12] Dong W, Xu C, Li C, Sun J, Zuo Y, Shi S, Cheng T, Guo J, Zhou S (2015) *ycf*1, the most promising plastid DNA barcode of land plants. Sci Rep 5:p.8348 10.1038/srep08348 PMC4325322 25672218

[13] Thomson AM, Vargas OM, Dick CW (2017) Comparative analysis of 24 chloroplast genomes yields highly informative genetic markers for the Brazil nut family (*Lecythidaceae*). bioRxiv:192112

[14] Krawczyk K, Nobis M, Myszczyński K, Klichowska E, Sawicki J (2018). Plastid super-barcodes as a tool for species discrimination in feather grasses (*Poaceae: Stipa*). Sci Rep.

[15] Wang S, Shi C, Gao LZ (2013). Plastid genome sequence of a wild woody oil species, Prinsepia utilis, provides insights into evolutionary and mutational patterns of *Rosaceae* chloroplast genomes. PLoS One.

[16] Choi KS, Chung MG, Park S (2016). The complete chloroplast genome sequences of three veroniceae species (*Plantaginaceae*): comparative analysis and highly divergent regions. Front Plant Sci.

[17] Bi Y, Zhang MF, Xue J, Dong R, Du YP, Zhang XH (2018). Chloroplast genomic resources for phylogeny and DNA barcoding: a case study on *Fritillaria*. Sci Rep.

[18] Dong W, Liu J, Yu J, Wang L, Zhou S (2012). Highly variable chloroplast markers for evaluating plant phylogeny at low taxonomic levels and for DNA barcoding. PloS One.

[19] Wyman SK, Jansen RK, Boore JL (2004). Automatic annotation of organellar genomes with DOGMA. Bioinformatics.

[20] Edgar RC (2004). MUSCLE: multiple sequence alignment with high accuracy and high throughput. Nucleic Acids Res.

[21] Kumar S, Stecher G, Li M, Knyaz C, Tamura K (2018). MEGA X: molecular evolutionary genetics analysis across computing platforms. Mol Biol Evol.

[22] Metsalu T, Vilo J (2015). ClustVis: A web tool for visualizing clustering of multivariate data using Principal Component Analysis and heatmap. Nucleic Acids Res.

[23] Tamura K, Nei M (1993). Estimation of the number of nucleotide substitutions in the control region of mitochondrial DNA in humans and chimpanzees. Mol Biol Evol.

[24] Potter D, Eriksson T, Evans RC, Oh S, Smedmark JEE, Morgan DR, Kerr M, Robertson KR, Arsenault M, Dickinson TA (2007). Phylogeny and classification of *Rosaceae*. Plant Syst Evol.

[25] Song Y, Dong W, Liu B, Xu C, Yao X, Gao J, Corlett RT (2015). Comparative analysis of complete chloroplast genome sequences of two tropical trees *Machilus yunnanensis* and *Machilus balansae* in the family Lauraceae. Front Plant Sci.

[26] Rohwer JG, Li J, Rudolph B, Schmidt SA, VWH LHW (2009). Is *Persea* (*Lauraceae*) monophyletic. Evidence from nuclear ribosomal ITS sequences. Taxon.

[27] Amar MH, Hassan AH, Biswas MK, Dulloo E, Xie ZZ, Guo WW (2014). Maximum parsimony based resolution of inter-species phylogenetic relationships in *Citrus* L. (Rutaceae) using ITS of rDNA. Biotechnol Biotechnol Equip.

[28] Guo C, Mcdowell IC, Nodzenski M, Scholtens DM, Allen AS, Lowe WL, Reddy TE (2017). Transversions have larger regulatory effects than transitions. BMC Genomics.

[29] Korotkova N, Nauheimer L, Ter-Voskanyan H, Allgaier M, Borsch T (2014). Variability among the most rapidly evolving plastid genomic regions is lineage-specific: implications of pairwise genome comparisons in *Pyrus* (Rosaceae) and other angiosperms for marker choice. PLoS One.

[30] Meng D, Xiaomei Z, Wenzhen K, Xu Z (2019). Detecting useful genetic markers and reconstructing the phylogeny of an important medicinal resource plant, *Artemisia selengensis* , based on chloroplast genomics. PLoS One.

[31] Wang J, Li C, Yan C, Zhao X, Shan S (2018). A comparative analysis of the complete chloroplast genome sequences of four peanut botanical varieties. PeerJ.

[32] Cao Y, Luo Q, Tian Y, Meng F (2017). Physiological and proteomic analyses of the drought stress response in *Amygdalus mira* (Koehne) Yü et Lu roots. BMC plant biology.

[33] Biswajit D, Ahmed N, Pushkar S (2011). *Prunus* diversity-early and present development. a review. Int J Bio Diverse Conserv.

[34] Yazbek M, Oh SH (2013). Peaches and almonds: phylogeny of *Prunus subg. Amygdalus* (Rosaceae) based on DNA sequences and morphology. Plant Syst Evol.

[35] Feng Y, Liu T, Wang XY, Li BB, Liang CL, Cai YL (2018). Characterization of the complete chloroplast genome of the Chinese cherry *Prunus pseudocerasus* (Rosaceae). Conserv Genet Resour.

